# Early-onset *COQ8B (ADCK4)* glomerulopathy in a child with isolated proteinuria: a case report and literature review

**DOI:** 10.1186/s12882-020-02038-7

**Published:** 2020-09-21

**Authors:** Shu-bo Zhai, Li Zhang, Bai-chao Sun, Yan Zhang, Qing-shan Ma

**Affiliations:** Department of Pediatrics Nephrology, First Hospital, Jilin University, Changchun, 130021 Jilin China

**Keywords:** Isolated proteinuria, Child, *COQ8B (ADCK4)* Glomerulopathy, CoQ10

## Abstract

**Background:**

Herein, a 3-year-old boy presented with hidden-onset isolated proteinuria was reported. The disease was induced by *COQ8B* (previously termed *ADCK4*) compound heterozygous variants, including c.[271C > T] and c.[737G > A], which were inherited from his father and mother, respectively.

**Case presentation:**

The patient visited our clinic due to non-nephrotic range proteinuria for 3 months, but no obvious abnormality was detected in the vital signs or laboratory test results. Renal histopathology revealed mitochondrial nephropathy, which manifested as mild glomerular abnormalities under light microscope, together with mitochondrial proliferation and hypertrophy and crowded arrangement under electron microscope. As suggested by whole exome sequencing, the patient inherited the *COQ8B* compound heterozygous variants from both of his parents who showed normal phenotype. After literature review, it was confirmed that one of the variant site (c.[271C > T]) had not been reported among the East Asian populations so far.

**Conclusions:**

Steroid-resistant nephrotic syndrome and focal segmental glomerulosclerosis are the most common phenotypes and renal histopathological manifestations of *COQ8B* variant. Nonetheless, our case shows that such variant may have hidden and mild clinical manifestations at the early onset. Therefore, early diagnosis will help to identify children at the early disease stage who have opportunity to benefit from oral coenzyme Q10 supplementation.

## Background

Proteinuria is one of the most common causes that prompt children and adolescents to visit a pediatric nephrologist. As reported by one study involving 37,645 children, 2.3% children have developed persistent proteinuria [1]. Transient or orthostatic proteinuria is usually benign, while persistent proteinuria may represent an early renal disease [2]. In fact, proteinuria is an independent risk factor that induces renal disease progression [3]. However, under numerous circumstances, proteinuria fails to arouse enough attention, and no accurate treatment is applied until it comes to end-stage renal disease (ESRD). In this paper, a child presented with isolated proteinuria was reported, and his renal histopathology showed mitochondrial nephropathy. Upon examinations, the disease was identified to be induced by the compound heterozygous variants in *COQ8B* (*ADCK4*) gene, and the patient was diagnosed with *ADCK4*-associated glomerulopathy (*ADCK4*-GN).

*ADCK4*-GN is an autosomal recessive chronic kidney disease, which can be induced by allelic homogeneous variant or compound heterozygous variants of *ADCK4* gene [4]. Patients with *ADCK4*-GN mainly manifest as steroid-resistant nephrotic syndrome (SRNS) [5], and their renal histopathology mostly manifest as focal segmental glomerulosclerosis (FSGS) [6]. Although the morbidity of *ADCK4*-GN remains unclear so far, *ADCK4* variant has accounted for the most common monogenic disorder among the SRNS patients in China [7, 8].

Considering the hidden onset, rapid progression and poor prognosis of the disease, especially with ESRD as the outcome, early diagnosis plays an essential role in terminating disease progression by Coenzyme Q10 (CoQ10) supplementation at the early stage [9].

## Case presentation

A 3-year-old boy was admitted to our hospital due to isolated proteinuria for 3 months. The patient did not receive routine urine test until he got pharyngitis. Notably, the quantity of proteinuria once reached the nephrotic range (0.93 g/24 h, 53 mg/kg), which then decreased to between 0.59 g/24 h and 0.62 g/24 h after the recovery of infection. During the entire course of disease, there was no edema. The patient was treated with Chinese herbal medicines at the local hospital although no definite diagnosis was made. However, proteinuria was not improved after nearly 3 months of treatment.

Both of his parents were healthy and provided normal results in routine urine test. In addition, they denied any positive family history of nephropathy. No obvious abnormality was detected in the prenatal examinations, and his vital signs and systematic physical examinations were normal. The total quantity of proteinuria was within the non-nephrotic range (590 mg/24 h, 33 mg/kg), which was dominated by albuminuria (522.90 mg/24 h). Besides, there was no abnormality in the low molecular weight proteinuria (LMWP) or the urine calcium-to-creatinine ratio, with no hematuria. Laboratory investigations revealed mild hypoalbuminemia (35.7 g/L), no hypercholesterolemia (4.52 mmol/L) and normal glomerular filtration rate (eGFR, 189.54 ml/min). Moreover, the patient did not have any significant illnesses, including hepatitis B, tuberculosis, IgA vasculitis or systemic lupus erythematosus. Besides, both ASO and EBV-IgM were negative. Renal ultrasound revealed normal echogenicity (left kidney, 72 mm × 32 mm, right kidney, 68 mm × 27 mm).

Clinically, the patient presented with isolated proteinuria, which was identified as glomerular proteinuria, while both the low molecular weight proteinuria and urine calcium-to-creatinine ratio were normal. Therefore, renal tubular diseases, such as Dent disease, were ruled out. Furthermore, there was no evident evidence of nephrotic syndrome since the patient had neither nephrotic proteinuria nor hypercholesterolemia, regardless of the mild hypoalbuminemia. Moreover, there was no evidence of secondary nephritis, including purpura nephritis, lupus nephritis or hepatitis B-related nephropathy. Based on the above findings, the patient was treated with angiotensin converting-enzyme inhibitor (ACEI) before renal biopsy, hoping to improve proteinuria.

**Fig. 1:**
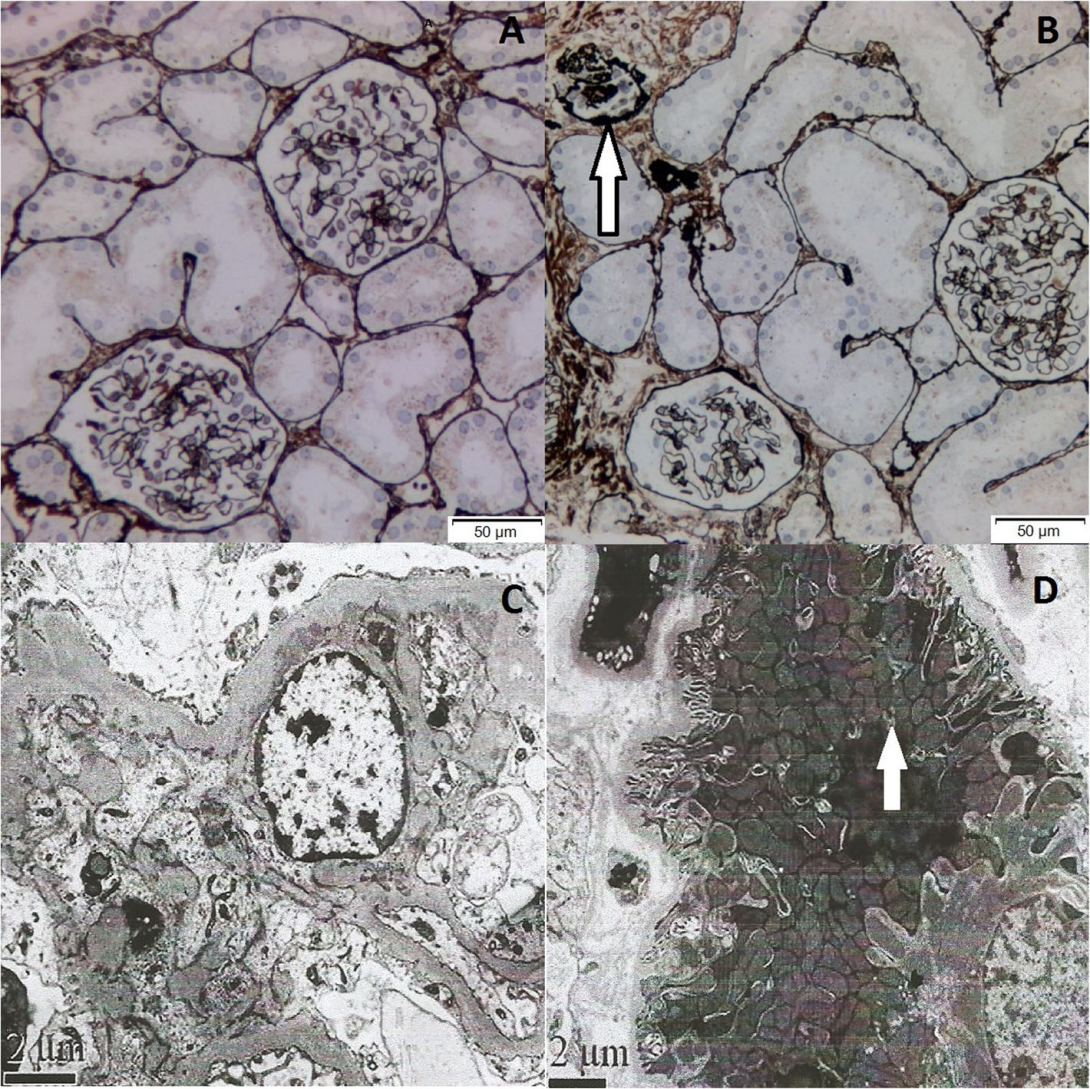
Renal histology of the patient. **a** Most glomeruli show no abnormality by PASM staining. **b** Among the 32 glomeruli, one reveals global glomerulosclerosis (white arrow heads). (PASM× 200). (Method: Light microscope: OLYMPUS, BX51. Image acquisition system: OLYMPUS, DP72.) (**c**) EM shows mild hyperplasia of mesangial cells and mesangial matrix. (× 10,000) (**d**) Proliferation and hypertrophy of mitochondria and crowded arrangement are observed on the lumen plane of renal tubules. (× 8000) (Method: Electron microscope: JEM1230, Japan JEOL Company. Camera: Model 832 digital image transmission instrument (CCD Camera), America Gatan Company)

Renal histopathology under light microscope (LM) showed that, one glomerulus turned sclerosis, while the other thirty one glomeruli exhibited minor glomerular abnormalities (Fig. 1b). Immunofluorescence was negative. Besides, electron microscope (EM) observation revealed mitochondrial proliferation and hypertrophy, as well as crowded arrangement in the lumen plane of renal tubules (Fig. 1d).

**Fig. 2:**
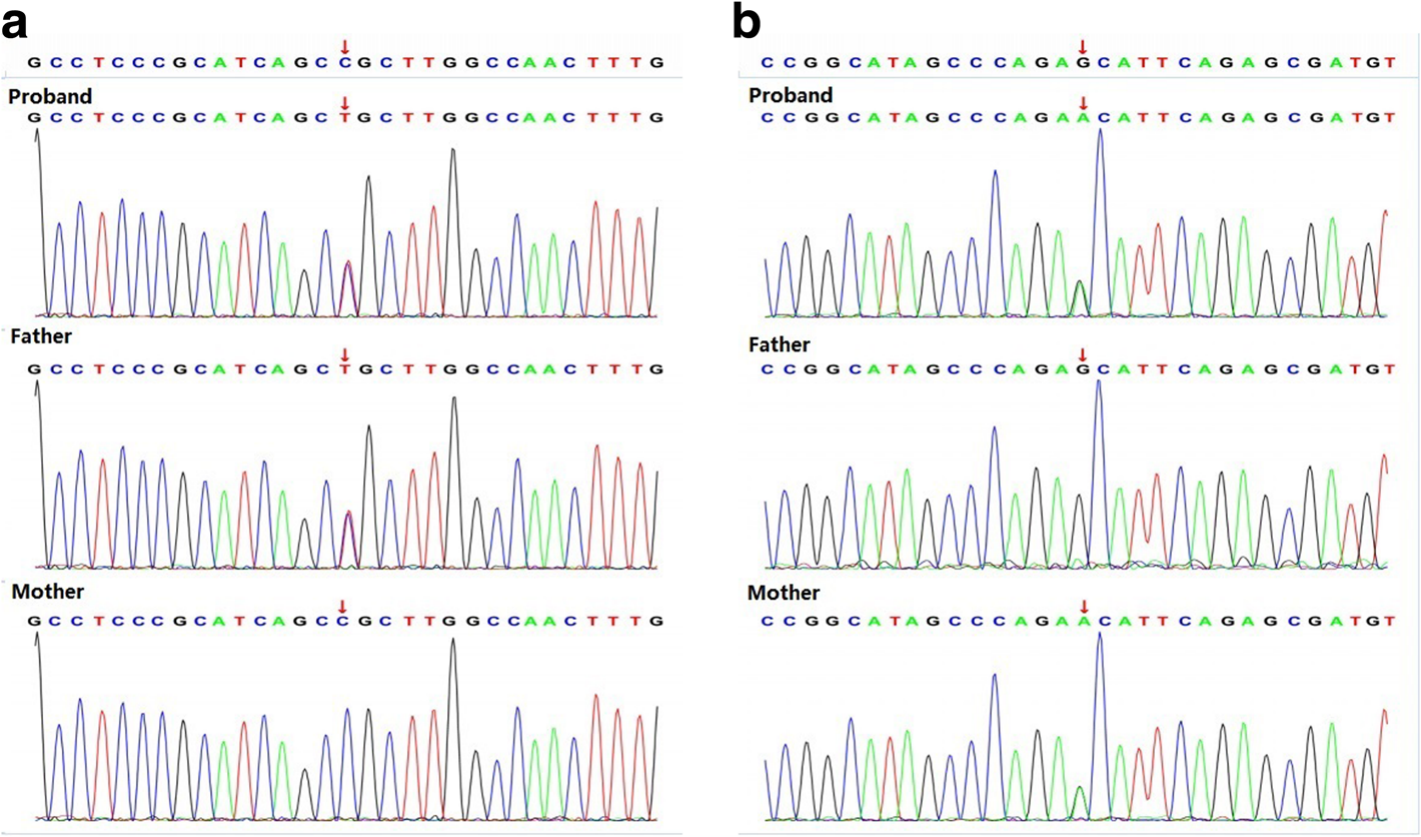
Genomic analysis. WES results of the patient and his parents indicate that our patient has 2 variants in the *COQ8B* gene: (**a**) c.[271C > T], p.(Arg91Cys), exon 4, chr19:41219990, inherited from his father; (**b**) c.[737G > A], p.(Ser246Asn), exon 9, chr19:41209508, inherited from his mother. Mitochondrial variants are not found

**Fig. 3:**
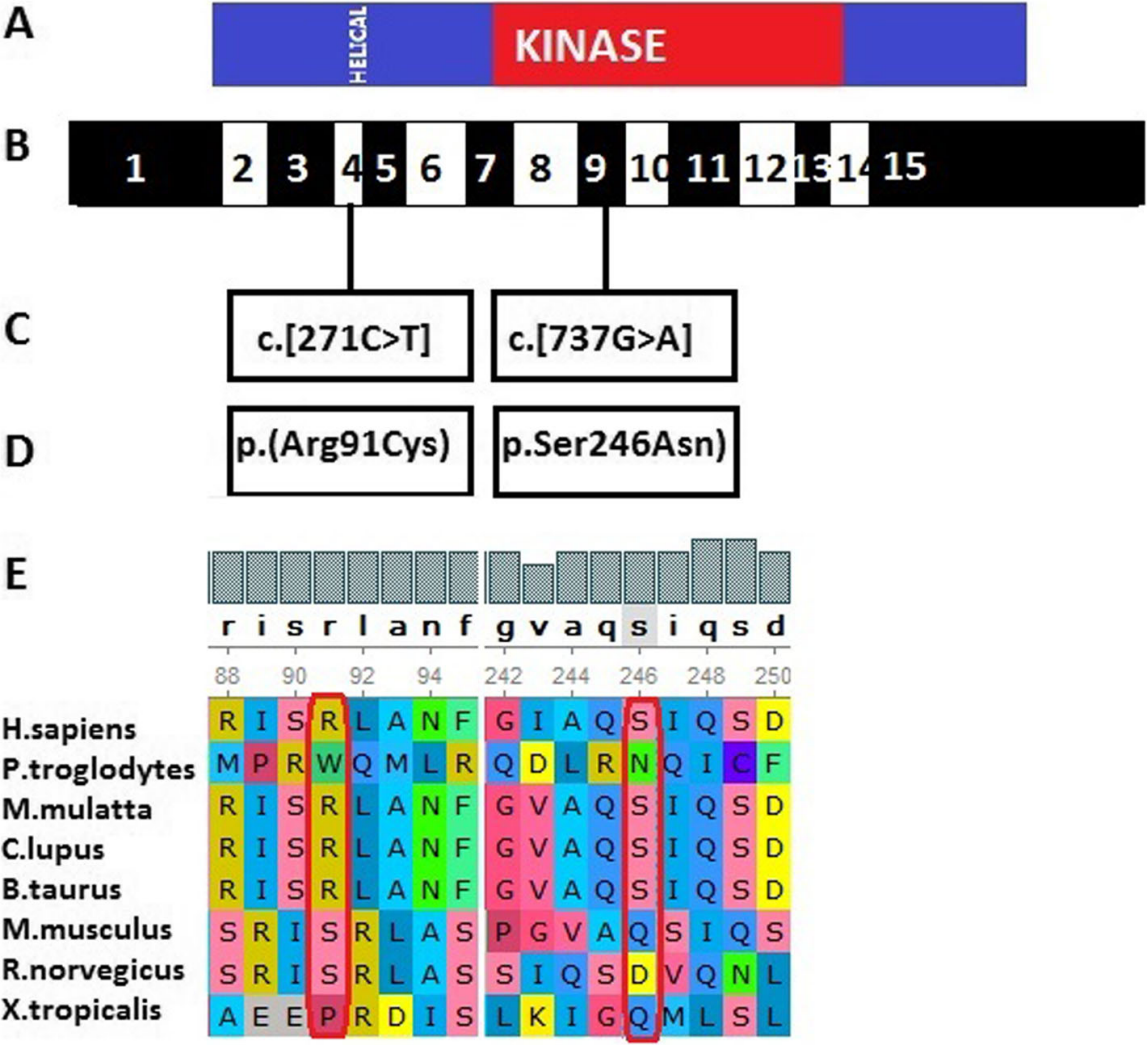
**a** Domain structure of ADCK4. The helical and kinase domains are depicted by colored bars in relation to encoding exon position. **b** Exon structure of human ADCK4 cDNA. ADCK4 contains 15 exons. **c** Variants of the ADCK4 gene. **d** Amino acid changes. **e** For the 2 missense variants conservation across evolution of altered amino acid residues is shown

As confirmed by Whole exome sequencing (WES) analysis, there were compound heterozygous variants in the *COQ8B* gene inherited from both of his parents. Of them, the variant (c.[271C > T]), which had not been reported among Asians, was inherited from his father (Fig. 2a) (According to GnomAD, this variant was presented in heterozygous state and detected in three individuals in Europeans. (https://gnomad.broadinstitute.org/variant/19-41219990-G-A?dataset=gnomad_r2_1.). Meanwhile, the missense variant (c.[737G > A]), which had been reported previously, was inherited from his mother (Fig. 2b). The pathogenicity of both variants was analyzed, as displayed in Table 1. The online Polyphen2 and SIFT software were used for the interpretation of the missense sequence variants. In silico (computational) prediction, these variants were predicted to be PROBABLY DAMAGING using the Polyphen2 tool (http://genetics.bwh.harvard.edu/pph2/). However, the prediction result was deleterious using SIFT tool (http://sift.jcvi.org). MAF of 0.00 for the c.[271C > T] variant was shown in population data, including dbSNP, 1000 Genomes Project, ExAC (East Asia), ExAC (South Asia), while in GnomAD, this variant was presented in heterozygous state and detected in three individuals in Europeans. MAF for the c.[737G > A] variant varied between different databases, as displayed in Table 1. According to ACMG, the pathogenicity of the sequence variant c.[271C > T] was of uncertain significance (PP3). The proband’s father with the same heterozygous variant had normal phenotype. Similarly, the pathogenicity of the sequence variant c.[737G > A] was also of uncertain significance (PP3), and his mother also showed normal phenotype. Notably, the sequence variant c.[271C > T] was located at exon 4, which coded the helical domain, whereas the sequence variant c.[737G > A] was located at exon 9 and coded the kinase domain. The amino acids encoded by the two above-mentioned sequence variants were of evolutionary conservation (Fig. 3). The variants in the proband were compound heterozygotes, which were consistent with the pathogenesis of autosomal recessive (AR) compound heterozygous genetic diseases. Moreover, the segregation of phenotypes and genotypes of the proband was consistent with that of his family members. Neither other nephrotic syndrome-related genes nor any copy number variations were detected. Mitochondrial detection also came to negative results. Combined with the typical renal histopathological findings, the diagnosis of *ADCK4*-GN was made, which was derived from the compound heterozygous sequence variants of the *COQ8B(ADCK4)* gene.

**Table 1:** Genomic analysis

Gene	Chr	Nucleic acid(Exon)	amino acid	RS	Pathogenicity (ACMG)	Proband	Father	Mother
*COQ8B*	chr19:41219990	c.[271C > T](exon 4)	p.(Arg91Cys)	rs75497 5339	Uncertain significance: PP3^a^	Het	Het	Wild type
	chr19:41209508	c.[737G > A](exon 9)	p.(Ser246Asn)	rs2008 41458	Uncertain significance: PP3^b^	Het	Wild type	Het
^a^(sift,Polyphen2_HDIV,Polyphen2_HVAR,PROVEAN,MutationTaster,M- CAP,REVEL,GERP,phyloP20way,phastCons20way)								
**Population data**						**Computational and predictive data**		
**Database**	**dbSNP**	**1000 Genomes Project**	**ExAC (East Asia)**	**ExAC (South Asia)**	**GnomAD (East Asia)**	**Polyphen2**	**SIFT**	
MAF	Not included	Not included	0.00	0.00	0.00	PROBABLY DAMAGING	Deleterious	
^b^(sift,Polyphen2_HDIV,Polyphen2_HVAR,MutationTaster,M-CAP,GERP,phyloP20way,phastCons20way)								
**Population data**						**Computational and predictive data**		
**Database**	**dbSNP**	**1000 Genomes Project**	**ExAC (East Asia)**	**ExAC (South Asia)**	**GnomAD (East Asia)**	**Polyphen2**	**SIFT**	
MAF	0.000088	0.0024	0.0013	0.00	0.018	PROBABLY DAMAGING	Deleterious	

^a^(sift,Polyphen2_HDIV,Polyphen2_HVAR,PROVEAN,MutationTaster,M- CAP,REVEL,GERP,phyloP20way,phastCons20way)

^b^(sift,Polyphen2_HDIV,Polyphen2_HVAR,MutationTaster,M-CAP,GERP,phyloP20way,phastCons20way)

Given the potential neurological involvement, further neurological system examinations were performed, including physical examinations and imaging examinations. The proband presented with neither hypotonia nor psychomotor delay. No abnormality was detected in brain magnetic resonance imaging (MRI) or electroencephalogram (EEG), and there was neither visual loss nor sensorineural hearing loss.

ACEI treatment was thus terminated, and the child was given treatment with CoQ10 (15 mg/kg·d) since then. Proteinuria and eGFR were persistently monitored, which showed that proteinuria gradually decreased during the 3-month-follow-up period (0.33 g/24 h, 18.9 mg/kg in the third month). In the fifth month, proteinuria decreased to 0.28 g/24 h, and eGFR was within the normal range.

## Discussion and conclusions

*ADCK4*-GN is a disease related to mitochondrial dysfunction. Generally, mitochondrial dysfunction can be caused by variants in either mitochondrial DNA (mtDNA) or nuclear DNA (nDNA) genes that encode proteins involved in the mitochondrial function [10]. It has been identified that, variants of *COQ8B (ADCK4)* gene can cause CoQ10 deficiency and mitochondrial nephropathy [11]. The affected patients may present with isolated renal diseases or accompanying extrarenal symptoms. For instance, one Japanese paper first reported a patient with Crohn’s disease (CD) combined with ADCK4-GN [12]. CoQ10, also known as the ubiquinone, is a vital part of the mitochondrial respiratory chain, which participates in the beta-oxidation of fatty acids and the synthesis of pyrimidine nucleoside; in addition, it serves as a cofactor for mitochondrial dehydrogenases [13]. CoQ10 is synthesized ubiquitously through the multienzyme complex on the inner mitochondrial membrane, in which at least 15 genes are involved [14] It seems that two genes have a regulatory role, including *COQ8* (and its human counterparts *COQ8A/ADCK3* and *COQ8B/ADCK4*) that encodes a putative kinase [15]. *COQ8B* interacts with the components in the CoQ10 biosynthesis pathway, and patients harboring *COQ8B* variants have reduced intracellular CoQ10 contents. Furthermore, it is reported that *COQ8B* is expressed in glomerular podocytes and partially localized in podocytic mitochondria as well as the podocytic processes in rat kidneys and cultured human podocytes [16]. Variants of *COQ8B* sequence participate in the effacement of podocytic process and the disorganization of filtration slit, which contributes to albuminuria and even nephrotic syndrome.

Variants in *COQ8B* sequence frequently cause SRNS with variable neurological involvement [17]. Typically, nephrotic syndrome constitutes the common etiology of proteinuria. About 10–20% pediatric patients with nephrotic syndrome do not achieve sustained remission after glucocorticoid therapy or additional immunosuppressive therapy, and finally progress into ESRD [18]. With the development of gene detection technology for pathogenic variants, genes that result in SRNS have attracted wide attention. Currently, at least 50 genes have been identified to lead to monogenic SRNS [19, 20]. A Chinese study [7] presents the spectrum of mutations among the Chinese children with SRNS, in which the most common mutated gene is *COQ8B* (*ADCK4*), followed by *NPHS1*, *WT1*, and *NPHS2*. Another recent Chinese cohort study investigates the genetic spectrum of renal disease among 1001 Chinese children based on the multicenter registration system. The results demonstrate that the mutation of *COQ8B* (*ADCK4*) gene is the major cause of SRNS [8]. The reported cases with *COQ8B* (*ADCK4*) are often diagnosed with SRNS, with FSGS as the renal histopathology [21], and such patients may even develop renal failure. Our patient clinically presented with isolated proteinuria without edema, along with normal renal function but not extrarenal symptoms. Typically, proteinuria may be of glomerular or tubulointerstitial origin [2]. For our case, tubulointerstitial proteinuria was first considered, because of his persistent proteinuria without edema. The 24-h urine protein excretion tests showed that albuminuria was dominant, with no low molecular weight proteinuria detected. Besides, his renal histopathology under EM showed mitochondrial nephropathy. Fortunately, the patient did not progress to FSGS. Using the WES technology, the *COQ8B* compound heterozygous variants were identified in the patient. *COQ8B* gene is located on human chromosome 19q13.2, which encodes the *COQ8B* protein. There are three functional regions in the *COQ8B* protein, including a helical domain, an ABC1 domain and a kinase domain, and it interacts with members of the CoQ10 biosynthesis pathway, including COQ6. The *COQ8B* compound heterozygous variants were identified in our patient, of which, the variant c.[271C > T] was inherited from his father, while c.[737G > A] was inherited from his mother. The amino acid residue 246 is located in the kinase domain, whereas the residue 91 is in the helical domain. The proteins affected by the above-mentioned sequence variants were conserved. Literature review was conducted to examine the phenotype and genotype of *ADCK4* variants in children (Table 2), which suggested that allelic homogeneous variants or compound heterozygous variants of *ADCK4* gene were the causes of disease. Similarly, the nephropathy of our proband was caused by the compound heterozygous variants of *ADCK4* gene. Different from the reported SRNS and FSGS, our proband showed mild phenotype and renal histopathology.

**Table 2:** Phenotype and genotype of *CoQ8B(ADCK4)* variants in children

References	Family- individual	Nucleotide alteration	Amino acid change	Ethnic group	Phenotype	Renal histopatholgy	Treatment
Ashraf et al [11] (2013)	1–1	c.101[G > A] (het) c.[954_956dup] (het)	p.(Trp34*) p.(Thr319dup)	European	SRNS	FSGS	PNL, tacrolimus, renal transplantation
	2–1	c.[532C > T] (homo)	p.(Arg178Trp)	Arab	SRNS	GS	renal transplantation
	2–2	c.[532C > T] (homo)	p.(Arg178Trp)	Arab	SRNS	FSGS	renal transplantation
	3–1	c.[645delT] (het) c.[1430G > A] (het)	p.(Phe215Lfs*14) p.(Arg477Gln)	Algerian Algerian	SRNS	FSGS	renal transplantation
	3–2				SRNS	FSGS	renal transplantation
	4–1	c.[857A > G] (het) c.[1447G > T] (het)	p.(Asp286Gly) p.(Glu483*)	ND (no data)	SRNS	FSGS	CsA, renal transplantation
	4–2				SRNS	FSGS	ND
	4–3				SRNS	FSGS	ACEI
	5–1	c.[958C > T] (homo)	p.(Arg320Trp)	Tunisian	SRNS	FSGS	ACEI, renal transplantation
	5–2				SRNS	ND	No treatment
	6–1	c.[1027C > T] (homo)	p.(Arg343Trp)	Moroccan	SRNS	ND	ND
	6–2				SRNS	FSGS	PNL
	7–1	c.[1199-1200insA] (homo)	p.(His400Nfs*11)	Turkish	SRNS	FSGS	PNL, ACEI, CP-R, CoQ10
	8–1	c.[1356-1362del] (homo)	p.(Gln452Hfs)	Indian	SRNS	FSGS	CsA, ACEI
	8–2				SRNS	FSGS	ACEI
Li et al [22] (2015)	1–1	c.[625C > G] (homo)	p.(Asp209His)	Chinese	Proteinuria	FSGS	CoQ10
Korkmaz et al [21] (2016)	26patients From 12families	c.[293 T > G]	p.(Leu98Arg)	Turkey	SRNS	FSGS	Immunosuppressive therapy; CoQ10
		c.[929C > T]	p.(Pro310Leu)	Turkey	SRNS	FSGS	
		c.[1493-1494CC > AA]	p.(Ala498Glu)	Turkey	SRNS	FSGS	
		c.[1339dupG]	p.(Glu447Glyfs*10)	Turkey	SRNS	FSGS	
Wang et al [7] (2017)	1–1	c.[241G > T] (het) c.[1468C > T] (het)	p.(Glu81*) p.(Arg490Cys)	Chinese	Proteinuria	FSGS	ND
	2–1	c.[448C > T] (het) c.[748G > C] (het)	p.(Arg150*) p.(Asp250His)	Chinese	SRNS	FSGS	ND
	3–1	c.[532C > T] (het) c.[748G > C] (het)	P.(Arg178Trp) p.(Asp250His)	Chinese	SRNS	Sclerosing Glomerulonep-hritis	ND
	4–1	c.[737G > A] (homo)	p.(Ser246Asn)	Chinese	SRNS	FSGS	ND
	5–1	c.[737G > A](homo)	p.(Ser246Asn)	Chinese	Proteinuria	MsPGN	ND
	6–1	c.[748G > C](homo)	p.(Asp250His)	Chinese	NS	ND	ND
	7–1	c.[748G > C] (homo)	p.(Asp250His)	Chinese	SRNS	FSGS	ND
	8–1	c.[748G > C] (het) c.[1093C > G] (het)	p.(Asp250His) p.(Gln365Glu)	Chinese	Proteinuria	FSGS	ND
Feng C et al [23] (2017)	1–1	c.[748G > C] (het) c.[532C > T] (het)	p.(Asp250His) P.(Arg178Trp)	Chinese	Proteinuria	ND	CoQ10
	2–1	c.[625C > G] (het) c.[614C > T] (het)	p.(Asp209His) p.(Ser205Asn)	Chinese	SRNS	ND	PNL, tacrolimus, CoQ10
Song X et al [24] (2017)	1–1	c.[737G > A] (het) c.[748G > C] (het)	p.(Ser246Asn) p.(Asp250His)	Chinese	Proteinuria	FSGS+GS	PNL, tacrolimus, ACEI,CoQ10
	2–1	c.[737G > A] (homo)	p.(Ser246Asn)	Chinese	SRNS	Global abandonment	PNL, HD, PD, CoQ10
	3–1	c.[737G > A] (homo)	p.(Ser246Asn)	Chinese	SRNS	FSGS	PNL, PD, renal transplantation
	4–1	c.[748G > C] (homo)	p.(Asp250His)	Chinese	SRNS	MsPGN	PNL, tacrolimus,MMF, CoQ10
	4–2	c.[748G > C] (homo)	p.(Asp250His)	Chinese	SRNS	EndPGN	PNL, HD, PD, CoQ10
	5–1	c.[748G > C] (homo)	p.(Asp250His)	Chinese	Proteinuria	FSGS+GS	ACEI,CoQ10, PD
	6–1	c.[551A > G] (het) c.[737G > A] (het)	p.(Asp184Gly) p.(Ser246Asn)	Chinese	Proteinuria	FSGS+GS	PNL, CsA, tacrolimus, RTX, CTX, PD, CoQ10
	7–1	c.[737G > A] (homo)	p.(Ser246Asn)	Chinese	Proteinuria	FSGS	PNL, CoQ10
Park E et al [6] (2017)	1–1	c.[449G > A] (het) c.[759C > A] (het)	p.(Arg150Gln) p.(Asn253Lys)	Korean	Proteinuria	FSGS	ND
	1–2	c.[449G > A] (het) c.[759C > A] (het)	p.(Arg150Gln) p.(Asn253Lys)	Korean	Proteinuria	FSGS	ND
	2–1	c.[737G > A] (het) c.[759C > A] (het)	p.(Ser246Asn) p.(Asn253Lys)	Korean	Proteinuria	FSGS	ND
	3–1	c.[737G > A] (homo)	p.(Ser246Asn)	Korean	Proteinuria	FSGS	ND
	4–1	c.[737G > A] (het) c.[1468C > T] (het)	p.(Ser246Asn) p.(Arg490Cys)	Korean	Proteinuria	FSGS	ND
	5–1	c.[737G > A] (homo)	p.(Ser246Asn)	Korean	Proteinuria	FSGS	CoQ10
Lolin K et al [25] (2017)	1–1	c.[649G > A] c.[748G > T]	p.(Ala217Thr) P.(Asp250Tyr)	Belgian	Proteinuria	FSGS	CoQ10
Yang J et al [16] (2018)	1–1	c.[625C > G] (het) c.[918G > T] (het)	p.(Asp209His) p.(Cys306X)	Chinese	Proteinuria	FSGS	CoQ10
Atmaca M et al [26] (2019)	1–1, 1–2	c.[1199dupA] (homo)	p.(His400Glnfs*11)	European	Proteinuria	ND	CoQ10
	2–1	c.[1199dupA] (homo)	p.(His400Glnfs*11)	European	Proteinuria	ND	CoQ10
	3–1	c.[1199dupA] (homo)	p.(His400Glnfs*11)	European	Proteinuria	ND	CoQ10
	4–1	c.[293 T > G] (homo)	p.(Leu98Arg)	European	Proteinuria	ND	ACEI, ARB, CoQ10
Yang Z et al [27] (2019)	1–1	c.[748G > C] (het) c.1041G > T (het)	p.(Asp250His) p.(Cys347*)	Chinese	Proteinuria	MGA	ND

*ND* No data or not do, *GS* Global glomerulosclerosis, *het* Heterozygous, *homo* Homozygous, *PNL* Prednisolone, *CsA* Cyclosporine A, *CP-R* Cyclophosphamide resistant, *FSGS* Focal segmental glomerulosclerosis, *MsPGN* Mesangial proliferative glomerulonephritis, *EndPGN* Endoproliferative glomerulonephritis, *MGA* Minor glomerular abnormalities

Primary coenzyme Q10 deficiency is considered as the only treatable mitochondrial disorder, since these patients can respond to the given oral coenzyme Q10 supplementation [28]. Early study considers that, oral coenzyme Q10 may stop the progression of encephalopathy, but it is not beneficial for the evolution of renal disease associated with this deficiency [29, 30]. Recently, Ashraf et al. [11] reported a SRNS patient with homozygous *ADCK4* frameshift mutation who had partial remission following CoQ10 treatment. Korkmaz et al. [21] reported two patients with early stage *ADCK4*-GN who demonstrated improved proteinuria during the 6-week CoQ10 supplementation. In addition, Atmaca et al. [31] examined the efficacy of CoQ10 supplementation in treating eight patients with *ADCK4*-GN, and found that proteinuria was significantly improved, whereas eGFR was preserved. Feng et al. [23] reported two children with proteinuria renal disease related to *ADCK4* mutation, of them, case 1 was a 9-month-girl with non-nephrotic range proteinuria who achieved full response to CoQ10 therapy, while case 2 was diagnosed with SRNS who showed the renal pathology of FSGS and had no response to CoQ10 supplementation. Another paper suggests that the early administration of CoQ10 is important for mitigating the renal symptoms of CoQ10 nephropathy [28]. Our patient received CoQ10 supplementation when he was 3 years old and the proteinuria gradually decreased by 52.5% during the 5-month follow-up period. Yet, such a follow-up period is still too short to evaluate the effects, which is one of the shortcomings in this paper. Nonetheless, in a long-term follow-up study that applies CoQ10 supplementation in patients with ADCK4 variants diagnosed during the asymptomatic period, it is discovered that, CoQ10 treatment is effective on reducing proteinuria, which seems to be renoprotective [26]. Combined with these previous reports and our data, it is implied that the early recognition of *ADCK4* mutation and the early CoQ10 supplementation to patients are important for patients with full or partial response to CoQ10 supplementation and benign prognosis.

Notably, rapid progression to ESRD is common in most cases, even though in our case, the patient presented with mild phenotype at the time of diagnosis. Besides, renal biopsy and WES have greatly facilitated the early diagnosis, which can also avoid misdiagnosis and the use of toxic drugs. Early CoQ10 supplementation achieves positive effects, therefore, early diagnosis will help to identify children at the early disease stages who are eligible for accurate CoQ10 therapy with benign prognosis.

## Data Availability

The datasets used and analyzed in this study are available from the corresponding author on reasonable request.

## Abbreviations

ADCK4: AarF domain-containing kinase 4
ADCK4-GN: ADCK4-associated glomerulopathy
MGA: Minor glomerular abnormalities
WES: Whole exome sequencing
CoQ10: Coenzyme Q
LM: Light microscope
EM: Electron microscope
eGFR: Estimated glomerular filtration rate;
SRNS: Steroid-resistant nephrotic syndrome
FSGS: Focal segmental glomerulosclerosis
LMWP: Low molecular weight proteinuria
ACEI: Angiotensin converting-enzyme inhibitor
ESRD: End-stage renal disease
NGS: Next generation sequencing
